# First Molecular Evidence of Seewis Virus in Croatia

**DOI:** 10.3390/life13122359

**Published:** 2023-12-18

**Authors:** Petra Svoboda Karić, Barbara Anđelić Dmitrović, Stella Mrmić, Antonia Paić, Linda Bjedov, Zrinka Štritof, Josip Margaletić, Ivan-Christian Kurolt

**Affiliations:** 1 Research Department, University Hospital for Infectious Diseases “Dr. Fran Mihaljević”, Mirogojska 8, 10000 Zagreb, Croatia; 2 Department of Forest Protection and Wildlife Management, Faculty of Forestry, University of Zagreb, Svetošimunska 25, 10000 Zagreb, Croatia; 3 Department of Microbiology and Infectious Diseases with Clinic, Faculty of Veterinary Medicine, University of Zagreb, Heinzelova 55, 10000 Zagreb, Croatia

**Keywords:** orthohantavirus, insectivores, shrew-borne, *Sorex araneus*, *Neomys milleri*, zoonosis

## Abstract

Orthohantaviruses are mainly carried and transmitted by wild rodents, although during the last decade, they have also been identified in multiple species of shrews and moles. Orthohantavirus, *Orthohantavirus seewisense* (Seewis virus, SWSV), first detected in Switzerland in a single *Sorex araneus* (Eurasian common shrew) specimen, has been further described in several European countries, including Croatia’s neighboring Slovenia and Hungary. Croatia is a well-known endemic region for several zoonotic agents including three different orthohantaviruses: *Orthohantavirus puumalaense* (PUUV), *Orthohantavirus dobravaense* (DOBV), and *Orthohantavirus tulaense* (TULV). In this study, nine shrews were tested and SWSV RNA was detected in liver, lung, and kidney belonging to two shrews (22.22%), one collected on Medvednica mountain in Zagreb County, and the other in the Stara Gradiška area in lowland Croatia. The phylogenetic analysis of the complete S segment’s open reading frame (ORF) and partial L-segment revealed that the Croatian sequences, when compared to sequences from the adjacent geographic regions, form a specific genetic lineage. Two SWSV-positive shrew species—*Sorex araneus* and *Neomys milleri* (Mediterranean water shrew)—were identified using barcode-based sequence analysis. Therefore, the SWSV detection in *N. milleri* throughout the course of this study is seen as a rare find in this shrew species. To our knowledge, this is the first molecular and phylogenetic analysis of SWSV in Croatia.

## 1. Introduction

Orthohantaviruses are negative-stranded RNA viruses distributed worldwide, belonging to the order *Bunyavirales*, family *Hantaviridae*. They are mainly carried and transmitted by wild rodents, but they have also been identified in multiple species of shrews and moles [1]. Each orthohantavirus is well-adapted to mostly one specific rodent or insectivore host. Even though several orthohantaviruses can induce diseases in humans upon inhalation of virus-contaminated aerosols, it is still unclear whether shrew-borne orthohantaviruses are pathogenic to humans. The first shrew-borne virus, *Thottimvirus thottapalayamense* (Thottapalayam virus, TPMV), was isolated from *Suncus murinus* (Asian house shrew) caught 1964 in southern India. Questions were raised on whether TPMV was naturally harbored by the insectivore host or represented a spillover from a rodent host [2]. In the paper by Song et al. [3], the first genetic and phylogenetic analysis of a new orthohantavirus—*Orthohantavirus seewisense* (Seewis virus, SWSV)—is presented, with partial genome sequences amplified from the lung tissue of a single *Sorex araneus* (Eurasian common shrew) specimen.

The report from Song et al. [3] corroborates reports of hantaviral antigens in tissues of several shrew species and the common mole from the European Russia and the former Yugoslavia [4,5], which went largely unnoticed decades earlier.

SWSV has been described in several European countries [6,7,8,9,10,11,12], including Hungary and Slovenia, two countries bordering Croatia [6,13]. Different SWSV strains exhibit geographically specific genetic variations, indicating a long history of orthohantavirus–host co-evolutionary adaptation [6]. Although *S. araneus* appears to be its main host, SWSV has been found in several other shrew species, including the former *N. anomalus* species group [7,8,10,14].

Croatia is a well-known endemic region for orthohantaviruses and other zoonotic agents [15]. So far, three different hantaviruses have been proven in Croatia: *Orthohantavirus puumalaense* (Puumala virus, PUUV) and *Orthohantavirus dobravaense* (Dobrava virus, DOBV) with medical importance [16,17], as well as *Orthohantavirus tulaense* (Tula virus, TULV) in rodents only and with uncertain medical importance [18]. However, we are lacking the molecular characterization and phylogenetic analysis of SWSV in Croatia.

## 2. Materials and Methods

### 2.1. Shrew Specimen Sampling

Nine shrew specimens in total were gathered from sampling locations in continental Croatia (mountainous and lowland) between 2013 and 2017 as part of routine rodent monitoring (Table 1) along linear transects, using snap traps (the permits for field sampling were in agreement and oversight of the Croatian forests Ltd., Public Institution “National park Plitvice lakes”, Public Institution “Nature park Medvednica” and Ministry of Economy and Sustainable Development). Guidelines by Gannon et al. [19] were followed. Shrew specimens were morphologically examined, and consequently, their organs were separated. Prior to further analysis, the samples were kept at −80 °C.

### 2.2. RNA Extraction, cDNA Synthesis and Amplification of S, M, and L Segments

Nucleic acids were extracted from shrew tissue samples (liver, lung, intestine, kidney or spleen tissue) using TriPure Isolation Reagent (Roche Applied Science, Penzberg, Germany) following the manufacturer’s instructions upon performing homogenization using TissueLyser II (Qiagen, Hilden, Germany). cDNA was synthesized using the M-MLV Reverse Transcriptase (Promega, Madison, WI, USA) according to the manufacturer’s protocol. Nested PCR reactions were performed using previously published primers [3,8,9,20,21] in order to amplify the S, M and L segments (Supplementary Table S1). The PCR amplicons were visualized on 2% agarose gels and purified by the AccuPrep PCR/Gel Purification Kit (Bioneer, Daejeon, Republic of Korea) or through the enzymatic purification of PCR amplicons with exonuclease 1 and alkaline phosphatase (New England Biolabs, Ipswich, MA, USA).

Using nested primers for the L segment and multiple primers to cover the entire S segment’s open reading frame (ORF), or partial M segment (Supplementary Table S1), direct sequencing of the purified PCR amplicons was performed using the BigDye Terminator v3.1 Cycle Sequencing Kit (Thermo Fisher Scientific-LifeTechnologies, Waltham, MA, USA) on 3500 Genetic Analyzer (Thermo Fisher Scientific-Applied Biosystems, Waltham, MA, USA). The attempt to recover the M segment genomic sequences using primers [3,9,21] resulted in poor sequencing.

### 2.3. Sequence Data Analysis and Phylogenetic Inference

The obtained sequences of S and L segments were aligned and assembled using Clone Manager v9 (Sci Ed Software LLC., Westminster, CO, USA) or BIOEDIT v.7.2 (Gene Codes Corporation, Ann Arbor, MI, USA) [22], respectively. The previously published SWSV sequences were accessed and obtained via GenBank (National Center for Biotechnology, NCBI, Bethesda, MA, USA) and aligned with S and L segments sequences in MEGA11 [23] using MUSCLE [24]. The alignments were quality-checked in Mesquite ver. 3 [25]. The Bayesian analysis was conducted using MrBayes v.3.2.7 [26] on the CIPRES Gateway [27] with optimal substitution models determined by jModelTest v. 2.1.6 [28]. The two runs with four Metropolis-coupled Monte Carlo Markov chains were performed for 10,000,000 generations, and trees were sampled every 1000 generations. The first 25% of sampled trees were deleted as burn-in after the effective sample was confirmed with Tracer v.1.7.2. [29], and a 50% majority-rule consensus tree was constructed, with nodal values representing the posterior probabilities. The maximum likelihood (ML) analysis was carried out on the CIPRES Gateway [27] as well using RAxML-HPC ver. 8.2.12 [30] with a GTRGAMMA model.

### 2.4. The Genetic Identification of Shrews

The genetic identification of shrews was achieved by amplifying the barcoding regions of the cytochrome b (*CytB*) [31] and the cytochrome c oxidase subunit I (*COI*) genes [32]. Direct sequencing of the purified PCR amplicons was performed with the forward and reverse primers for both genes, as described above. The identity of the shrew species was determined through a comparison of the acquired sequences to those that had been previously deposited in GenBank using the Basic Local Alignment Search Tool [33].

## 3. Results

Out of nine shrews, SWSV RNA was identified in two, using L segment sequencing (22.22%) (Table 1). SWSV RNA was identified in the lung and liver tissues of *S. araneus*, in addition to the kidney tissue of one *N. milleri* specimen (Table 1). Shrew species were confirmed using mitochondrial phylogenetic markers (Table 1).

The near-complete S-segment sequence was amplified from a single liver tissue sample (1525 bp). Such a 1525-bp-long sequence comprises the entire ORF of the S segment. The sequence was stored in the European Nucleotide Archive (ENA) under accession number OY759148. The pairwise comparison of the novel nucleotide sequence from Croatia showed 86.8% similarity to the SWSV mp70 strain (Acc no: EF636024). The deduced amino acid (aa) sequence with a pairwise comparison of aa sequence showed 96.7% similarity to the SWSV mp70 strain.

The partial SWSV L segment sequences (347 bp, nucleotide (nt) 2967–3313 of the SWSV prototype mp70 strain, Acc no: EF636026) were successfully amplified for positive shrews. Sequences were stored in the GenBank and/or ENA under the following accession numbers KP742969.1, OY759149, OY759150. The pairwise comparison of the novel nucleotide sequences from Croatia showed 72.8% similarity to the SWSV mp70 strain. The deduced aa sequence with the pairwise comparison of aa sequences showed 98.26% similarity to the SWSV mp70 strain.

**Figure 1:**
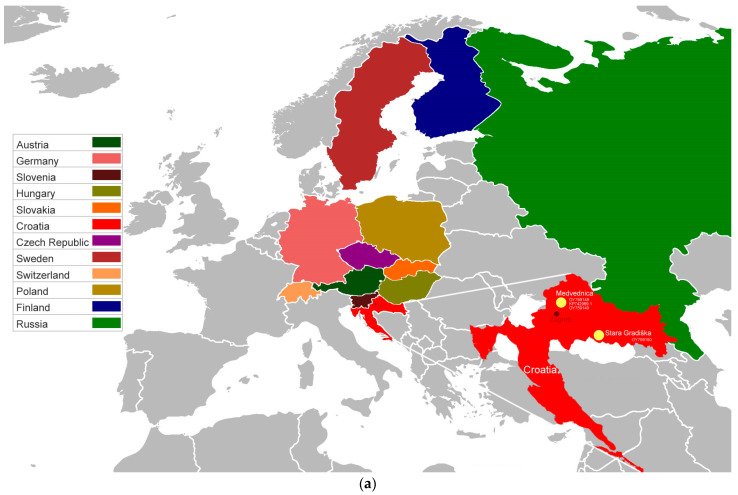

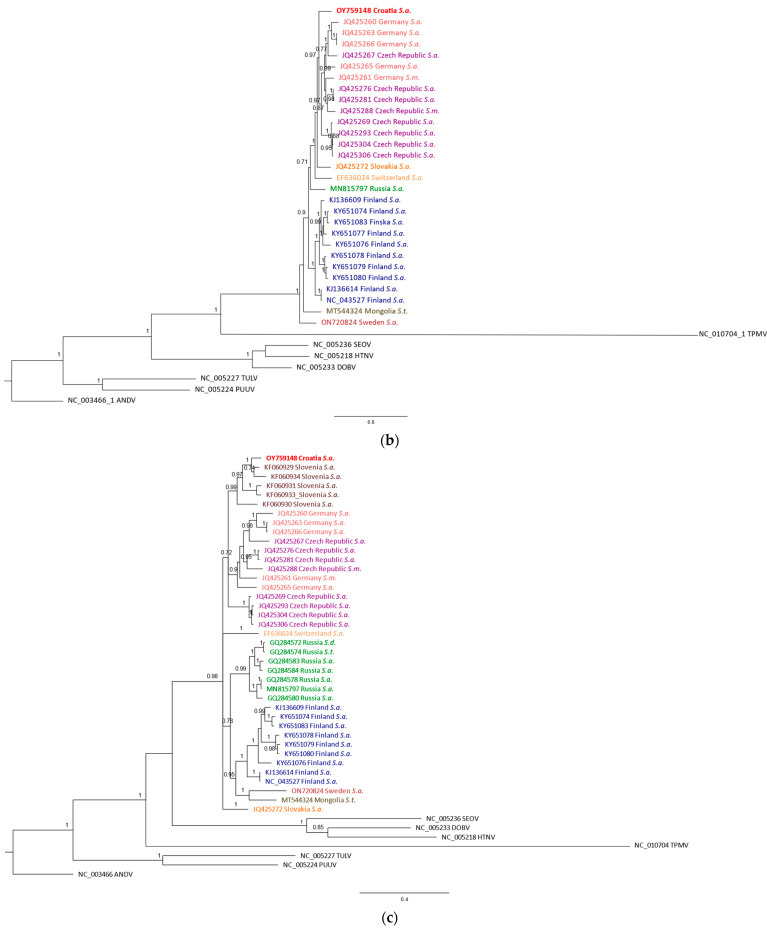

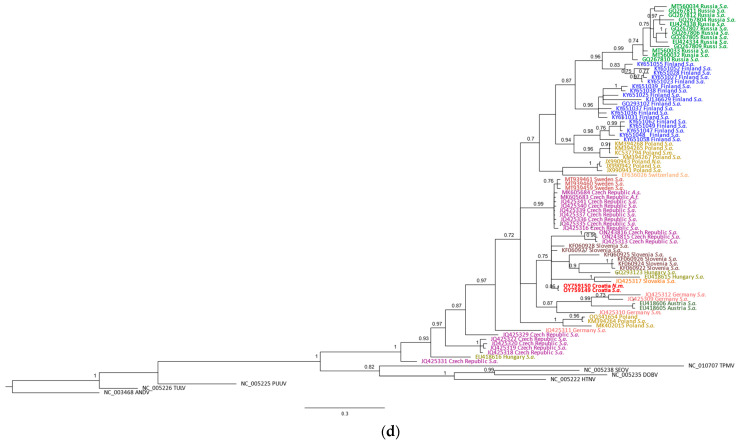
A color-coded geographic map showing the country of origin of *Orthohantavirus seewisense* (SWSV) sequences (**a**). Phylograms based on complete S-segment’s ORF (**b**), partial S-segment (**c**), and partial L-segment sequences (**d**) of SWSV. The phylogenetic trees were inferred using MrBayes v.3.2.7 [26]. The numbers on the branches represent posterior probabilities (≥0.7). The GTR + I + G was applied as the optimal nucleotide substitution model for both the S and L segment. The final dataset comprised 1290 or 834 positions altogether for the S, and 258 positions for the L segment, respectively. Sequences of SWSV strains are color-coded according to the country of origin and annotated with GenBank and ENA accession numbers. Viral detection in the specific animal taxa is indicated by the abbreviations: *A.f.*—*Apodemus flavicollis*, *A.s*.—*Apodemus sylvaticus*, *N.a.*—*Neomys anomalus*, *N.m.*—*Neomys milleri*, *S.a.*—*Sorex araneus*, *S.d.*—*Sorex daphaenodon*, *S.m.*—*Sorex minutus*, *S.t.*—*Sorex tundrensis*. Sequences from this study are highlighted in red. The sequence KP742969.1 was excluded from phylogenetic analyses due to its lower coverage compared to the other L segment sequences from this study. The tree was annotated in FigTree v1.4.4 [34], and the visualization was completed in Inkscape. The *Orthohantavirus andesense* (ANDV), *Orthohantavirus puumalaense* (PUUV), *Orthohantavirus tulaense* (TULV), *Orthohantavirus dobravaense* (DOBV), *Orthohantavirus hantanense* (HTNV), *Orthohantavirus seoulense* (SEOV), and *Thottimvirus thottapalayamense* (TPMV) sequences were included as outgroups.

The overall phylogenetic analysis (Figure 1) showed that our sequences formed a distinct genetic lineage, with posterior probabilities of 0.86–1. For the S segment’s complete ORF analysis (Figure 1b), Slovenian sequences were excluded due to incomplete coverage of sequences deposited in NCBI GenBank. Complete ORF phylogenetic analyses (Figure 1b) resulted in a better-resolved tree, which, when compared to analyses of partial sequences (Figure 1c,d), had higher posterior probability values. Analyses of partial sequences placed Croatian sequences as the most closely related to those from neighboring Slovenia and Hungary, as well as to those from Central Europe (Czech Republic and Slovakia). Our sequence clustered exclusively with Slovenian sequences in the partial S-segment-based phylogeny, and was proven to be most closely related to Slovenian Notranjsko-Kraska Podravska genetic lineage (Acc no: KF060929 and KF060934) (Figure 1c). The ML tree results support Bayesian-derived phylogeny (Supplementary Figure S1).

## 4. Discussion

As previously stated, SWSV has been reported in *S. araneus* from neighboring countries [6,13]. In addition, high infection rates with every second animal being infected have been described in shrews in Finland [6], and moles in Poland and France for *Mobatvirus novaense* (Nova mobatvirus) [14,35]. Therefore, the detection of SWSV in *S. araneus* in Croatia is not unexpected. *S. araneus* is a solitary animal, with extremely aggressive territorial behavior, which increases the possibility of acquiring and transmitting orthohantaviruses through wounds [3]. Additionally, we found SWSV in *N. milleri*. Previously, *N. milleri* had been considered as a subspecies of *N. anomalus*. However, *N. anomalus* and *N. milleri* have been described as distinct species in a recent study by Igea et al. [36]. *N. milleri* has a broader geographic range over several European countries, while *N. anomalus* is found throughout the Iberian Peninsula and is endemic to the region. Only two cases—a study by Gu et al. [14] (Acc no: JX990943) and the incomplete L-segment sequence published in the GenBank databases (Acc no: EU418604)—have so far confirmed the presence of SWSV in the *N. anomalus* species group. Based on the locations of these findings (Poland and Austria) and the geographic distribution of *N. milleri* relative to *N. anomalus,* as reported by Igea et al. [36], these findings can be regarded as confirming the presence of the SWSV in *N. milleri*. Therefore, the detection of SWSV in *N. milleri* in this study turned out to be a rare find of this virus in this species. However, it is important to emphasize that in all three cases, only the short, conservative L segment sequences were detected, the longest being 412 bp. Maybe these are indications of a spillover, given this and the fact that our repeated attempts to amplify and sequence the S segment in the case of SWSV from *N. milleri* were unsuccessful. We believe that additional research is required to conclusively determine if *N. milleri* acts as a true reservoir host of SWSV, or whether these are all occurrences due to a spillover [14]. As previously stated, SWSV has been detected in other shrew species, apart from *S. araneus*, *S. minutus*, *S. tundrensis*, *S. daphaenodon* and *N. milleri,* which might represent spillover events, as seen previously with some rodent-borne orthohantaviruses, or even additional reservoir hosts, due to a broad geographical distribution of SWSV [7,8,14]. Whether shrews shed orthohantaviruses is still uncertain. Due to insectivore populations being smaller in comparison to rodent populations, as well as their solitary lifestyles, the possibility of virus transmission to humans due to excretion exposure is questionable [2]. Since *S. araneus* is the primary host of SWSV and is found in Croatia’s continental region, SWSV may also be distributed there. The trapping of SWSV-positive *S. araneus* was at 990 m above sea level on Medvednica mountain, and positive *N. milleri* was at 90 m above sea level in lowland area near Stara Gradiška bordering Bosnia and Herzegovina (Figure 1a). This could lead to the conclusion that occurrences of SWSV-positive shrews can also be expected in Bosnia and Herzegovina. The difference in sampling habitats (mountainous vs. lowland), as well as the distance between trapping sites (cca 150 km), could contribute to the formation of genetically and geographically distinct SWSV clusters, as seen for PUUV and DOBV [37]. Also, these areas, located in continental Croatia, adjacent to the Croatian capital Zagreb, are known as recreational areas and endemic regions with an unusually high percentage of rodents positive for pathogenic orthohantaviruses [38].

Phylogenetic analyses of S and L segments (Figure 1b–d) revealed that the Croatian sequences form a distinct genetic lineage. Further, our sequences were most closely related to the previously described Slovenian, Hungarian, Slovakian, and Czech Republic sequences. Within sister groups, the analysis showed evidence of geographic clustering of the European SWSV sequences, with S-segment-based phylogeny further distinguishing the sequences among the adjacent ones, in terms of geographic distribution, and when compared to the L-segment-based phylogeny. Using partial sequences [13], in Slovenia, the presence of great genetic SWSV diversity with three strains was confirmed, and our S-segment sequence turned out to be most closely related to the Notranjsko-Kraska Podravska genetic lineage. In order to clarify whether there are more SWSV strains present in Croatia, further studies are needed. Our L-segment-based phylogeny results were consistent with those by Ling et al. (2018) [9], who report that our sequence (KP742969.1) is more closely related to sequences from Slovenia, Hungary, and Slovakia. The supporting L segment analysis, compared to the complete S segment’s ORF analysis, reflected the fact that this short region is insufficient and not informative enough to completely disclose the evolutionary history of this virus, and that more complete genome sequences are required. Despite this, a short L segment region was employed in this study because the databases (NCBI GenBank, ENA, and BV-BRC: Bacterial and Viral Bioinformatics Resource Center) had the highest representation of such short sequences up to 347 bp. In addition, it is worth noting that, due to the lack of complete S segment sequences in databases, it was very challenging to utilize the phylogeny of the complete S segment.

Due to the small body size of shrews, their specific dietary requirements/needs and high metabolism [39], they unfortunately decay quickly in snap traps on trapping sites. Thus, obtaining viral sequences from shrew tissue samples is challenging. A similar event occurred in a study by Gu et al. [14] when 50% of shrews were found dead in live traps despite having been checked every four hours.

So far, there have been more than 10 novel and genetically distinct orthohantaviruses identified in shrews and moles (for the list, see Kuhn et al. 2023) [40]. These newfound orthohantaviruses from geographically widespread regions are genetically more diverse than those harbored by rodents. Analyses have shown that orthohantaviruses first appeared in moles and shrews in Asia before appearing in rodents, considering the largest orthohantavirus diversity in Asian moles and shrews and their basal position on phylogenetic trees [41,42,43,44]. These findings suggest that the evolutionary history of orthohantaviruses is far more complex than previously assumed.

This study, due to having a complete S segment ORF and partial L segment, contributes to the goal of assembling near-complete SWSV genome sequences. However, poor M segment sequencing is unfortunately still the reason why SWSV may not be confirmed as an orthohantavirus species. This is due to the requirement to assess viruses for which there is coding-complete or near-complete sequence information for all three segments [40].

Further research, with more (complete) SWSV sequences derived from *S. araneus* and other shrew species, will contribute to knowledge about the uniqueness of SWSV as an orthohantavirus species, as well as possible SWSV strain genetic diversity in Croatia.

## Figures and Tables

**Table 1:** List of shrew specimens tested with corresponding species, trapping sites and years, and results of SWSV detection in examined organs.

Sample	Species ^1^	Sampling Site	Coordinates	Trapping Year	SWSV RNA ^2^	Organs Examined ^3^
M-1390	*Sorex araneus*	Medvednica	N45 54.453 E15 58.058	2013	KP742969.1, OY759148, OY759149	**lungs (L)**, kidney, **liver (S, L)**, intestines
M-2115		Koprivnica	N46 11.405 E16 50.826	2017		kidney, liver, spleen
M-528	*Neomys milleri* ^4^	Ivanić Grad	N45 38.134 E16 26.011	2003		lungs, kidney
M-1824		Stara Gradiška	N45 11.866 E17 09.268	2016		kidney, liver, spleen
M-1825		Stara Gradiška	N45 11.866 E17 09.354	2016	OY759150	**kidney (L)**, liver, spleen intestines
M-2150		Lipovljani	N45 22.044 E16 52.631	2017		lungs, kidney, liver, spleen
M-1971	*Neomys fodiens*	Sunja	N45 24.363 E16 43.719	2016		lungs, kidney, liver
M-1993	*Crocidura suaveolens*	Čakovec	N46 21.087 E16 23.277	2016		lungs, kidney, liver, spleen
M-1650	*Crocidura leucodon*	Plitvice	N44 52.186 E15 36.017	2014		lungs, kidney, spleen

^1^ Results for both mitochondrial markers, *COI* and *CytB*, coupled. ^2^ The corresponding sequence accession numbers are listed for SWSV-positive samples. ^3^ SWSV-positive organs are highlighted in bold, with letters in brackets indicating segments sequenced from each organ. ^4^ Although in some sources (including genetic databases, e.g., NCBI GenBank) *Neomys milleri* is still listed as a subspecies of *Neomys anomalus* Cabrera, 1907, *Neomys milleri* has been elevated to the species level and is no longer regarded as such.

## Data Availability

Sequences are deposited in GenBank under accession number KP742969.1 and in the European Nucleotide Archive under accession numbers OY759148-OY759150, and are available at https://www.ebi.ac.uk/ena/browser/view/ (accessed on 8 November 2023).

## Supplementary Materials

The following supporting information can be downloaded at: https://www.mdpi.com/article/10.3390/life13122359/s1, Table S1. Oligonucleotides for SWSV S, M, and L segment amplification. Figure S1. The ML trees based on complete S-segment’s ORF (a), partial S-segment (b), and partial L-segment sequences (c). The numbers on nodes represent bootstrap support values (≥70%). The final dataset comprised 1290 or 834 positions altogether for the S, and 258 positions for the L segment, respectively. The SWSV strains are color-coded according to the country of origin. The tip labels display the GenBank and ENA accession numbers. The tree was annotated in FigTree v1.4.4 [34], and the visualization was completed in Inkscape. The *Orthohantavirus andesense* (ANDV), *Orthohantavirus puumalaense* (PUUV), *Orthohantavirus tulaense* (TULV), *Orthohantavirus dobravaense* (DOBV), *Orthohantavirus hantanense* (HTNV), *Orthohantavirus seoulense* (SEOV), and *Thottimvirus thottapalayamense* (TPMV) sequences were included as outgroups.
