# Supplementary material for: First Molecular Evidence of Seewis Virus in Croatia
Source: Life (Basel). 2023 Dec 18;13(12):2359. doi: 10.3390/life13122359 (PMC10744651; doi:10.3390/life13122359)
Supplement: Supplementary file 1 [file life-13-02359-s001.zip › Supplementary Table s1.pdf]

Supplementary Table 1. Oligonucleotides for SWSV S, M, and L segment amplification.

Genomic target	Oligonucleotide name	Oligonucleotide sequence	Reference
**SWSV S segment**	SWSV-22-fw	GCATACTACGAAACAGAGAGC	Schlegel et al. 2012
	SWSV-22-962F	TGGGTMTTYGCRGGWGCACCTGA	Schlegel et al. 2012
	SWSV 3'end	TAGTAGTAKRCTCCYTRAARAG	Schlegel et al. 2012
	SWSV1590R	GTG TTT GAG GTA KTG GAG TG	Schlegel et al. 2012
	BESS1090R	ACAGTCTTTGATGCRATRATGG	Radosa et al. 2013
	SWSV S455R	TAAGCAAGATGGTGTCTACG	**This study**
**SWSV M segment**	BESM-12F	TCCGCAAGAAAAAGCCACAT	Radosa et al. 2013
	OSV697F	GGACCAGGTGCADCTTGTGAAGC	Song et al. 2007
	MR685709	GCTTTAGWAATYAAAAAACAATSTAC	Ling et al. 2014
	BESM-1225R	GCTTCACAGGATGCACCAGGGC	Radosa et al. 2013
	TM 1485R	CCAGCCAAARCARAATGT	Song et al. 2007
**SWSV L segment**	HAN-L-F1	ATGTAYGTBAGTGCWGATGC	Klempa et al. 2006
	HAN-L-F2	TGCWGATGCHACIAARTGGTC	Klempa et al. 2006
	HAN-L-R1	AACCADTCWGTYCCRTCATC	Klempa et al. 2006
	HAN-L-R2	GCRTCRTCWGARTGRTGDGCAA	Klempa et al. 2006
