# Supplementary figures and images for: First Molecular Evidence of Seewis Virus in Croatia
Source: Life (Basel). 2023 Dec 18;13(12):2359. doi: 10.3390/life13122359 (PMC10744651; doi:10.3390/life13122359)

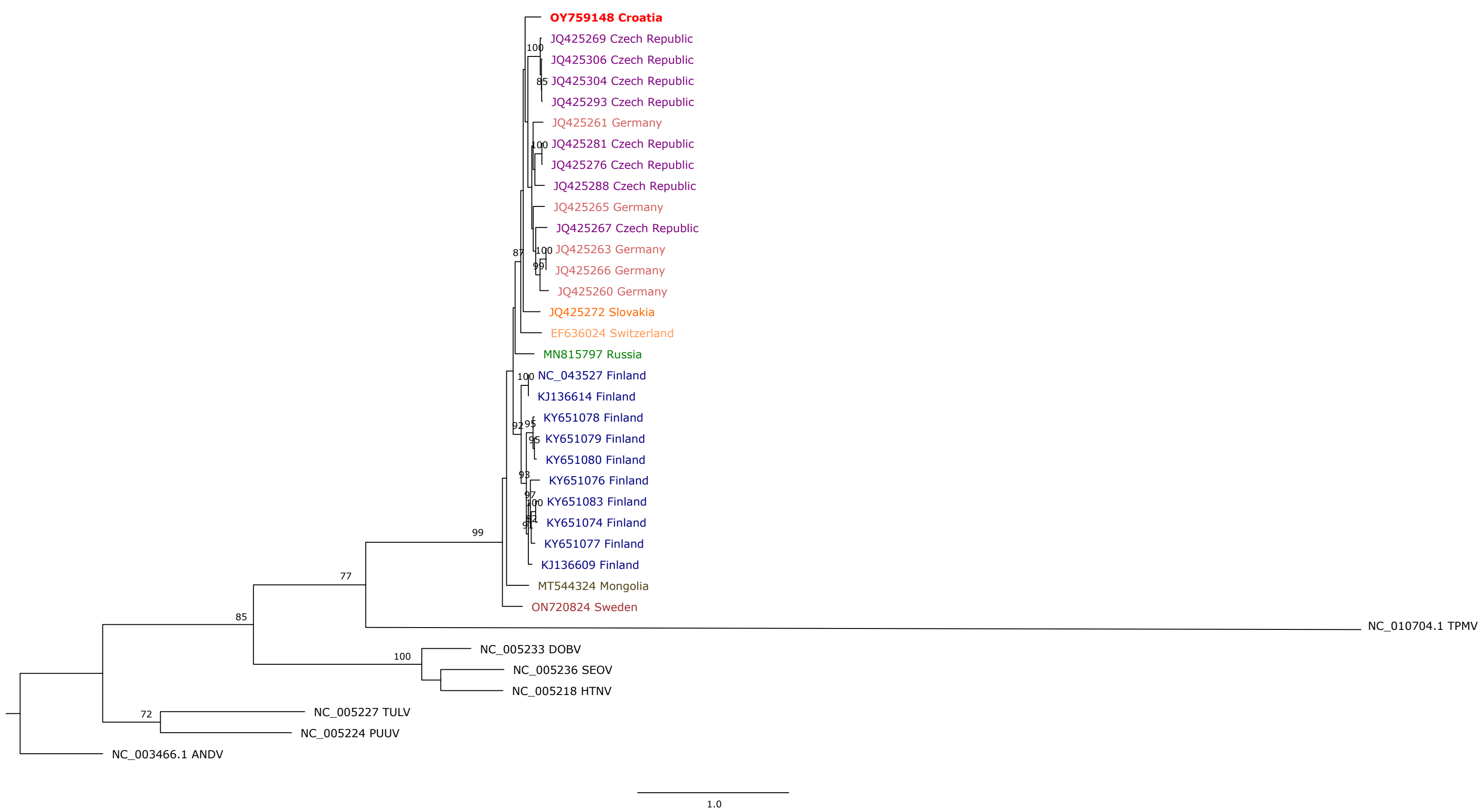

Supplement: Supplementary file 1 [file life-13-02359-s001.zip › Figure S1A.pdf]

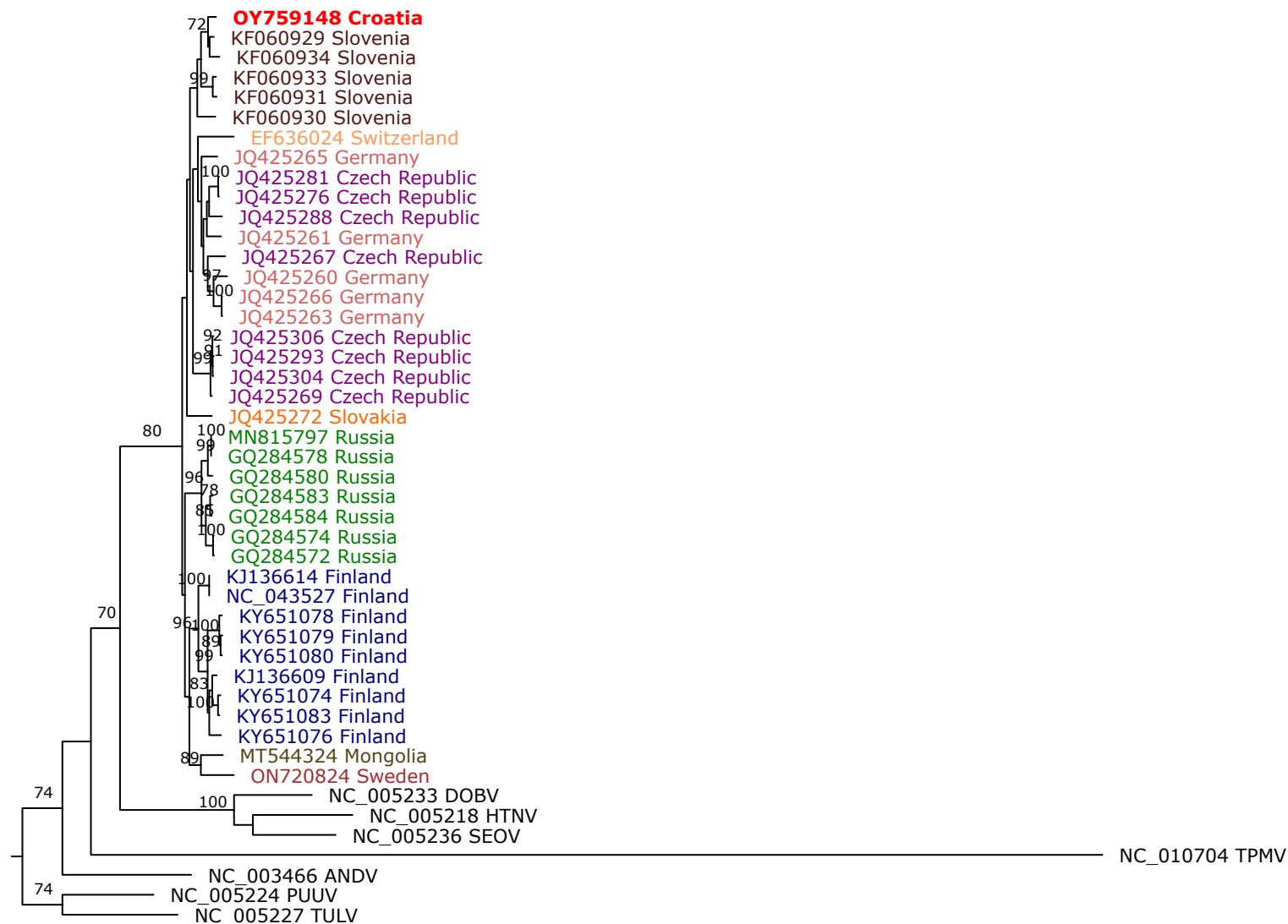

0.5

Supplement: Supplementary file 1 [file life-13-02359-s001.zip › Figure S1B.pdf]

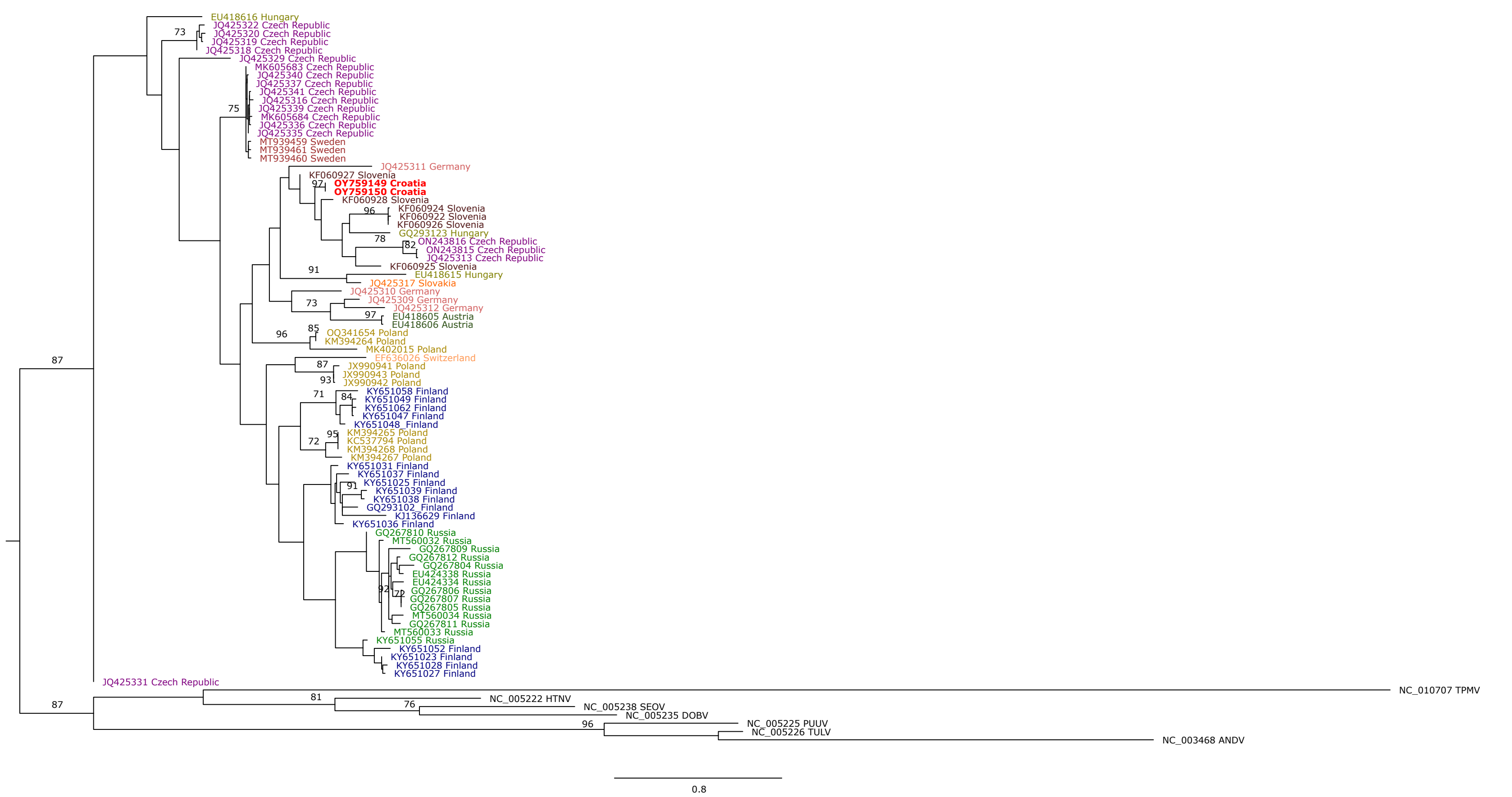

Supplement: Supplementary file 1 [file life-13-02359-s001.zip › Figure S1C.pdf]
